# Time Trends in Clinical Characteristics and Hospital Outcomes of Hospitalizations for Lung Transplantation in COPD Patients in Spain from 2016 to 2020—Impact of the COVID-19 Pandemic

**DOI:** 10.3390/jcm12030963

**Published:** 2023-01-26

**Authors:** Javier De Miguel-Diez, Rodrigo Jimenez-Garcia, Valentin Hernández-Barrera, David Carabantes-Alarcon, Jose J. Zamorano-Leon, Natividad Cuadrado-Corrales, Ricardo Omaña-Palanco, Francisco Javier González-Barcala, Ana Lopez-de-Andres

**Affiliations:** 1 Respiratory Care Department, Hospital General Universitario Gregorio Marañón, Instituto de Investigación Sanitaria Gregorio Marañón (IiSGM), Universidad Complutense de Madrid, 28007 Madrid, Spain; 2 Department of Public Health and Maternal & Child Health, Faculty of Medicine, Universidad Complutense de Madrid, 28040 Madrid, Spain; 3 Preventive Medicine and Public Health Teaching and Research Unit, Health Sciences Faculty, Universidad Rey Juan Carlos, 28922 Alcorcón, Spain; 4 Servicio de Neumología, Hospital Clínico Universitario de Santiago de Compostela, Universidad de Santiago de Compostela, 15706 Santiago de Compostela, Spain

**Keywords:** lung transplantation, COPD, hospital admissions, complications, in-hospital mortality, Spain

## Abstract

(1) Background: To examine the clinical characteristics and hospital outcomes of hospitalization for lung transplantation in COPD patients in Spain from 2016 to 2020; and to assess if the COVID-19 pandemic has affected the number or the outcomes of lung transplantations in these patients. (2) Methods: We used the Spanish National Hospital Discharge Database to select subjects who had a code for COPD (ICD-10: J44) and had undergone a lung transplantation (ICD-10 codes OBYxxxx). (3) Results: During the study period, 704 lung transplants were performed among COPD patients (single 31.68%, bilateral 68.32%). The absolute number of transplants increased with raising rates of 8%, 14% and 19% annually from 2016 to 2019. However, a marked decrease of −18% was observed from 2019 to year 2020. Overall, 47.44% of the patients suffered at least one complication, being the most frequent lung transplant rejection (24.15%), followed by lung transplant infection (13.35%). The median length of hospital stay (LOHS) was 33 days and the in-hospital-mortality (IHM) was 9.94%. Variables associated with increased risk of mortality were a Comorbidity Charlson Index ≥ 1 (OR 1.82; 95%CI 1.08–3.05) and suffering any complication of the lung transplantation (OR 2.14; 95%CI 1.27–3.6). COPD patients in 2020 had a CCI ≥ 1 in a lower proportion than 2019 patients (29.37 vs. 38.51%; *p* = 0.015) and less frequently suffered any complications after the lung transplantation (41.26 vs. 54.6%; *p* = 0.013), no changes in the LOHS or the IHM were detected from 2019 to 2020. (4) Conclusions: Our study showed a constant increase in the number of lung transplantations from 2016 to 2019 in COPD patients, with a drop from 2019 to 2020, probably related to the COVID-19 pandemic. However, no changes in LOHS or IHM were detected over time.

## 1. Introduction

Lung transplantation is the final therapeutic option for the management of patients with life-threatening, advanced lung disease, in whom other medical or surgical treatments have failed [1,2,3,4]. The rapid development in its efficacy in recent years has resulted in noticeable improvement in overall outcomes and prolonged survival [5].

Chronic obstructive pulmonary disease (COPD) is one of the most common indications for lung transplantation with about 35% of all procedures reported in the past decade [6]. Lung transplantation is associated with significant functional improvement, enhanced quality of life and survival advantage in rigorously selected patients with advanced COPD refractory to other treatments [7,8,9]. However, the shortage of suitable organs for transplantation limits the ability of patients who need to receive them and contributes to waiting list mortality. This issue is particularly important in lung transplantation because patients can receive either single lung transplantation or bilateral lung transplantation [9]. While both represent acceptable therapeutic options [10], the latter offers patients greater potential for long-term survival and avoids possible native lung complications in these patients [11].

Currently, there is limited literature on national trends in lung transplantation practices [12]. In Spain, there are studies indicating that hospitalization rates for lung transplantation increased significantly from 2001 to 2015 in COPD patients [13,14]. While there are few subsequent studies of trends, it appears that the COVID-19 pandemic caused a decrease in the mean number of lung transplantations in COPD and non-COPD subjects [15], as might have occurred in other countries [16,17]. Despite this, it does not seem like there has been a significant change in waiting list deaths compared with the same period in the preceding five years [18].

The objectives of our study were to examine the clinical characteristics and hospital outcomes of hospitalization for lung transplantation in COPD patients in Spain from 2016 to 2020; and second, to assess if the COVID-19 pandemic has affected the number or the outcomes of lung transplantations in these patients.

## 2. Materials and Methods

### 2.1. Design and Data Source

A retrospective, population-based observational study was conducted using the Spanish National Hospital Discharge Database (RAE-CMBD, Registro de Actividad de Atención Especializada-Conjunto Mínimo Básico de Datos (RAE-CMBD) (Register of Specialized Care–Basic Minimum Database)) as the database. The RAE-CMBD methodology description is available online [19]. The study period runs from 1 January 2016 to 31 December 2020.

The RAE-CMBD collects age, sex, dates of admission and discharge, discharge destination (home, deceased, social institution or voluntary discharge), up to 20 diagnosis and 20 procedures conducted during the hospitalization. The International Classification of Disease 10th version (ICD10) is used for coding since 2016.

### 2.2. Study Population and Study Variables

The study population included subjects who had a code for COPD (ICD-10: J44) in any position of the diagnostic fields and had undergone a lung transplantation (ICD-10 codes OBYxxxx, in any procedure field).

Single lung transplantation was considered if the ICD-10 codes were 0BYCxxx, 0BYDxxx, 0BYFxxx, 0BYGxxx, 0BYHxxx, 0BYJxxx, or 0BYKxxx and bilateral lung transplantation when codes were 0BYMxxx. Patients with heart and lung transplantation were excluded.

The presence of comorbidity has been assessed using the Charlson comorbidity index (CCI), excluding COPD, using the codes for ICD10 described by Sundararajan et al. and Quan et al. [20,21]. Likewise, regardless of the diagnostic position, the presence of pulmonary hypertension (ICD-10 codes: I27.0, I27.2x, I27.89) has been evaluated.

Procedures such as hemodialysis (ICD-10 codes: 5A1Dxxx), extracorporeal membrane oxygenation (ICD-10 codes: 5A15xxx) and tracheostomy (ICD-10 codes 0B11xxx) were identified in any of the procedure fields of the database.

The following complications derived from the lung transplantation were considered if recorded in any diagnostic position: Lung transplant rejection (ICD-10 code: T86.810), lung transplant failure (ICD-10 code: T86.811), lung transplant infection (ICD-10 code: T86.812), other complications of lung transplant (ICD-10 code: T86.818) and, unspecified complication of lung transplant (ICD-10 code: T86.819). We have considered patients to suffer “any complication of lung transplantation” if one or more of the aforementioned complications appeared in their diagnostic fields.

In addition, we have evaluated the presence of pneumonia (ICD10-codes: J13-J18) and ventilator-associated pneumonia (ICD-10 code: J95.851) as complications.

To identify possible infections caused by specific microorganisms we have searched for the following codes in the database: *Staphylococcus* bacteremia (A4101, A4102, A411, A412, A4901, A4902, B9561, B9562, B957, B958), *Streptococcus* bacteremia (A400, A401, A403, A408, A409, A491, B950, B951, B953, B954, B955), Gram-negative bacteremia (A413, A4150, A4151, A4152, A4153, A4159, B961, B9620, B9621, B9622, B9623, B9629, B963, B964, B965), Fungemia (B376, B377, B409, B393, B394, B395, B399, B449), *Pseudomonas aeruginosa* infection (B96.5), *Aspergillus* infection (B44.xx), Candidiasis infection (B37.7) and Cytomegalovirus infection (B25.x).

Length of hospital stay (LOHS) was defined as the number of days between hospital admission and discharge. In-hospital mortality (IHM) was the proportion of deaths during the hospital admission for lung transplantation.

### 2.3. Statistical Analysis

We have performed a descriptive analysis calculating mean with standard deviation (SD) or median with inter quartile range (IQR) for continuous variables and proportions for categorical variables.

To assess changes over time we used Cochran-Armitage tests for categorical variables, and the test for linear trend or the Jonckheere-Terpstra test for continuous variable.

We compared categorical variables using the χ^2^ test and continuous variables with the t test or the Mann–Whitney test, as required.

We conducted a multivariable logistic regression model to identify which study variables were independently associated with IHM. The variables included in the model were those with a significant association in the bivariate analyses. Results are shown as odds ratios (ORs) with their 95% confidence interval (CI).

Stata version 14 (Stata, College Station, TX, USA) was used for the statistical analysis. A *p* value < 0.05 (2-sided) was considered significant.

### 2.4. Ethics Statement

The RAE-CMBD is owned by the Spanish Ministry of Health (SMH) and can be accessed upon request [22]. We sent the protocol for this investigation to the SMH that approved it and provided us with the anonymized database. According to Spanish legislation, written consent from the patients is not required as this is an administrative registry.

## 3. Results

**Figure 1:**
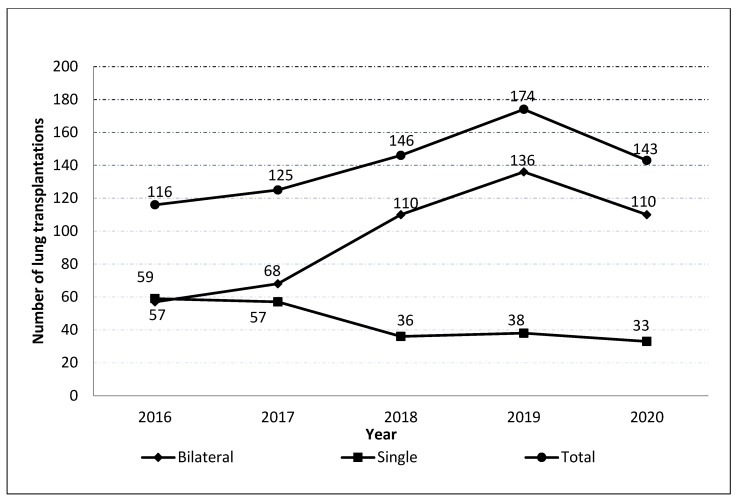
Number of lung transplantations in patient with COPD in Spain from 2016 to 2020, according to transplant type.

In Spain, during the period 2016-2020, a total of 704 lung transplantations were performed among subjects with an ICD-10 code for COPD. Of these, 481 were bilateral (68.32%) and the remaining single lung transplantations (31.68%). The number of lung transplantation by age and according to type can be seen in Figure 1.

Regarding the temporal evolution of lung transplantations in patients with COPD in Spain, the absolute number of transplants increased with raising rates of 8%, 14% and 19% annually from 2016 to 2019. However, a marked decrease of −18% was observed from 2019 to 2020. This decrement was larger for bilateral (136 to 110; −19%) than for single (38 to 33; −13%) lung transplantations.

Table 1 shows the main characteristics of patients with COPD who underwent a lung transplantation. Single lung transplantations decreased significantly overtime (50.86% in 2016 vs. 23.08% in 2020; *p* < 0.001) and therefore bilateral transplantations increased (49.14% in 2016 vs. 76.92% in 2020; *p* ≪ 0.001). Of the 704 transplants performed, 68.89% were in men. The mean age was 58.07 years (SD: 7.73). Of the transplanted patients 30.11% had at least one comorbidity (CCI ≥ 1). The median LOHS was 33 days and the IHM was 9.94%. As can be seen in the tables, none of these study variables changed significantly from 2016 to 2020.

The main comorbidities and procedures performed in patients with COPD who underwent a lung transplantation are presented in Table 2. Overall, pulmonary hypertension (16.76%), liver disease (8.66%), and diabetes (8.38%) were the most frequent comorbidities recorded. Only liver disease has changed significantly throughout the study period (4.31% in 2016 vs. 11.19% in 2020; *p* < 0.001). Regarding procedures, extracorporeal membrane oxygenation has increased significantly between 2016 and 2020 (10.34% in 2016 vs. 23.08% in 2020; *p* = 0.002). The proportion of patients requiring a tracheostomy was stable and conducted in 18.75% of patients from 2016 to 2020.

The prevalence of complications after lung transplantation and pathogens isolations are listed in Table 3. Overall, 47.44% of the patients who underwent a lung transplantation suffered at least one complication with this proportion increasing significantly from 2016 to 2020 (37.93% vs. 41.26%; *p* = 0.002). Lung transplant rejection was the most frequently coded complications (24.15%) followed by lung transplant infection (13.35%). Overall, the proportion of patients with pneumonia was 6.39% and 1.28% had ventilator-associated pneumonia.

The most isolated pathogens were *Staphylococcus* bacteremia (10.8%), Gram-negative bacteremia (6.96%) and *Pseudomonas aeruginosa* (6.39%). None of the pathogens analyzed changed its frequency over time.

The analysis of the IHM according to study variables is shown in Table 4. The IHM was not different beside the type of transplantation (single 10.76% vs. bilateral 9.56%; *p* = 0.621). Women had a not significantly higher IHM than men (12.33 vs. 8.87%; *p* = 0.155). The highest IHM was found among those aged under 40 years (19.05%) and the lowest in the 40–49 years group (5.77%), increasing afterwards as the age rose to reach 11.51% in the oldest age group (≥60 years). Patients with a CCI ≥ 1 had a significantly higher IHM (14.15%) when compared to those with a CCI = 0 (8.13%; *p* = 0.014). The IHM of those suffering any complication of the lung transplantation was twice the IHM for those not affected by these complications (13.47 vs. 6.76%; *p* = 0.003). The IHM has increased significantly overtime among those who underwent a single lung transplantation (8.47% in 2016 vs. 24.24% in 2020; *p* = 0.028) with no significant changes overtime for any other variable analyzed.

Table 5 shows the results of the multivariable logistic regression model to assess the factors independently associated with IHM in COPD patients after lung transplantation. After adjustment, a CCI ≥ 1, (OR 1.82; 95%CI 1.08–3.05) and suffering any complication of the lung transplantation (OR 2.14; 95%CI 1.27–3.6) were variables associated with increased risk of mortality. As can be seen in the table, the IHM showed no change from 2016 to 2020.

When the lung transplantations from 2019 to 2020 were compered, we observed that besides a reduction in the total number of transplants (−18%), the IHM and the LOHS were not statistically different. However, COPD patients in 2020 had a CCI ≥ 1 in a lower proportion than 2019 patients (29.37 vs. 38.51%; *p* = 0.015) and less frequently suffered any complications after the lung transplantation (41.26 vs. 54.6%; *p* = 0.013).

## 4. Discussion

In our study, we observed a progressive increase in the number of lung transplantations from 2016 to 2019, following the trend observed in previous years [14]. The International Society for Heart and Lung Transplantation has also recently reported that the number of lung transplant recipients with COPD has also increased by era (1992–2000, 2001–2009, and 2010–2018), although the proportion of COPD recipients compared to other transplant diagnoses has decreased over time. This trend reflects the expansion of acceptable donors and candidates, made possible by clinical and scientific advances [23].

However, our study showed a fall in the number of lung transplantation in 2020, which can be explained by the COVID-19 pandemic. In this way, hospital resource requirements, the availability and safety of deceased organ donors and the safety of transplant candidates and recipients, forced difficult decisions and changes to practice [16]. In fact, the biggest problem that the COVID-19 epidemic generated in the field of organ donation and transplantation was the consequence of the saturation of the health system and intensive care units. This led to a lack of COVID-19-free areas in hospitals, in which to guarantee the safety of patients once they were transplanted. In this way, in the worst moments, only ideal donors were considered, and the transplant carried out in patients with an emergency or extreme clinical severity. All of this led to an overall decrease in transplant activity in 2020 [24,25]. In a population-based observational study, in which national cohorts of consecutive kidney, liver, lung, and heart transplants from 22 countries were collected and validated, this same trend was observed. Lung transplantation was one of the most affected, after kidney transplantation and followed by liver and heart transplantation. The overall reduction in transplants during the observation time period translated into 48,239 years of life lost for patients on the waiting list [26]. Due to this, there is an urgent need to develop strategies to safely perform lung transplantation procedures and follow patients after treatment to limit direct and indirect impairment of the prognosis of potential and actual lung transplantation recipients [27].

Regarding type of transplant, single lung transplant was once considered the procedure of choice for COPD patients after management of the allograft was improved and the ventilation and perfusion mismatch was minimized [28]. However, preceding decades leading up to the modern day, the bilateral transplantation has gradually become the predominant surgical approach for these patients [29]. In the same line, we found a marked decrease in unilateral lung transplantations and an increase in bilateral ones. Factors that may influence this decision include: patients who underwent bilateral lung transplantation have improved exercise tolerance and pulmonary function tests compared with single lung transplantation, likely as a consequence of dynamic hyperinflation of the native lung; there is an increased risk of lung cancer in the native lung in patients with single lung transplantation due to chronic obstructive pulmonary disease; bilateral lung transplantation has been shown to improve median survival compared with single lung transplant, especially for younger recipients with COPD [30]. In the same way, a recent systematic review and meta-analysis has shown that bilateral lung transplantation appears to be associated with better long-term survival, better postoperative lung function, and less bronchiolitis obliterans syndrome compared to single lung transplantation [31]. It seems, therefore, that bilateral lung transplantation remains the preferred transplant type in selected patients with COPD yet to be challenged by more data [32]. Despite this, concern for waitlist mortality may drive the use of single lung transplantation despite better posttransplant survival in bilateral lung transplantation [9].

Neither sociodemographic nor clinical (LOHS and IHM) characteristics changed significantly from 2016 to 2020, despite the decrease in transplant activity detected in 2020. These results are in line with those described by other authors, who have described that outcomes after lung transplantation have remained largely unchanged over the last decade and continue to be worse than outcomes after other solid-organ transplantation [33].

Factors independently associated with IHM in COPD patients after lung transplantation in our study were a higher comorbidity and suffering any complication of the lung transplantation. Both factors have been previously described as predictors of IHM, in addition to others such as age [14]. However, Bello et al., in a retrospective, multicenter cohort study that collected data from 272 adults with lung transplantation, also demonstrated an independent association between pulmonary graft disfunction and SOFA (Sequential Organ Failure Assessment) score with early mortality after lung transplant [34].

Our study has several strengths and limitations that warrant discussion. Utilization of the RAE-CMBD provides us with an opportunity to analyze a large number of patients undergoing lung transplantation at a national level across a 5-year timeframe. However, a major limitation of our study is the reliance on accurate ICD-10 diagnostic and procedure coding and the possibility of underestimating or overestimating the numbers. On the other hand, our analyzes are based on the data provided by the RAE-CMBD, and they are limited by the lack of clinical data that could be relevant. Thus, for example, it is not possible to provide additional details about infections, but this information could be provided by the Spanish National Registry of Lung Transplantation [35]. It is also not possible to identify the kind of rejection or if more than one rejection episodes have occurred, since the complications are coded according to ICD-10. In addition, the RAE-CMBD is an anomyzed database, so it is not possible to compare lung transplant centers. Furthermore, given the cross-sectional nature of our analyses, no causality can be established. Nonetheless, administrative databases, with their limitations, have been used in the past in Spain and other countries to assess time trends and outcomes of different transplants [14,35,36,37,38,39,40,41,42,43,44]. In this regard, in the US, Fielding-Singh et al. evaluated the accuracy of the National Inpatient Sample (NIS) by comparing estimates of solid organ transplantation to known national transplant volumes from the Organ Procurement and Transplant Network, concluding that although NIS transplantation research may be limited by the inability to subgroup procedures by donor type, surgical procedure coding of solid organ transplantation, the NIS appears to be accurate and reliable for generating national estimates [45]. Finally, another limitation of this study is that there is no data available on the follow-up of patients after discharge.

## 5. Conclusions

In conclusion, our study showed a constant increase in the number of lung transplantations from 2016 to 2019 in COPD patients, with a drop from 2019 to 2020, probably related to the COVID-19 pandemic. However, no changes in length of hospital stay or IHM were detected over time. Further research is needed on the epidemiology of lung transplantation in COPD patients to better understand how to adapt to the increasing demand that lies ahead.

## Figures and Tables

**Table 1:** Clinical characteristics of patients with COPD who underwent a lung transplantation in Spain from 2016 to 2020.

		2016	2017	2018	2019	2020	TOTAL	*p*-Value Trend
Number of transplantations		116	125	146	174	143	704	
Type, n (%)	Single	59 (50.86)	57 (45.6)	36 (24.66)	38 (21.84)	33 (23.08)	223 (31.68)	<0.001
	Bilateral	57 (49.14)	68 (54.4)	110 (75.34)	136 (78.16)	110 (76.92)	481 (68.32)	
Men, n (%)		84 (72.41)	95 (76)	98 (67.12)	117 (67.24)	91 (63.64)	485 (68.89)	0.207
Age, mean (SD)		57.28 (7.73)	58.55 (5.98)	57.59 (7.7)	58.16 (8)	58.68 (8.73)	58.07 (7.73)	0.533
Age groups, n (%)	<40 years	5 (4.31)	2 (1.6)	5 (3.42)	7 (4.02)	2 (1.4)	21 (2.98)	0.414
	40–49 years,	6 (5.17)	6 (4.8)	15 (10.27)	13 (7.47)	12 (8.39)	52 (7.39)	
	50–59 years	52 (44.83)	52 (41.6)	54 (36.99)	61 (35.06)	47 (32.87)	266 (37.78)	
	≥60 years,	53 (45.69)	65 (52)	72 (49.32)	93 (53.45)	82 (57.34)	365 (51.85)	
CCI, mean (SD)		0.28(0.54)	0.31 (0.56)	0.34 (0.58)	0.48 (0.69)	0.36 (0.62)	0.36 (0.61)	0.056
CCI, n (%)	0	88 (75.86)	92 (73.6)	104 (71.23)	107 (61.49)	101 (70.63)	492 (69.89)	0.067
	≥1	28 (24.14)	33 (26.4)	42 (28.77)	67 (38.51)	42 (29.37)	212 (30.11)	
LOHS, median (IQR)		32.5 (20)	33 (24)	34 (31)	34 (20)	30 (18)	33 (21)	0.782
IHM, n (%)		11 (9.48)	10 (8)	17 (11.64)	17 (9.77)	15 (10.49)	70 (9.94)	0.897

CCI: Charlson comorbidity index. LOHS: Length of hospital stay. IHM: In-hospital mortality.

**Table 2:** Comorbidities included in the Charlson Comorbidity index, pulmonary hypertension and procedures on hospitalized patients with COPD who underwent lung transplantation in Spain from 2016 to 2020.

	2016	2017	2018	2019	2020	TOTAL	*p*-Value Trend
Myocardial infarction, n (%)	2 (1.72)	2 (1.6)	2 (1.37)	6 (3.45)	3 (2.1)	15 (2.13)	0.715
Congestive heart failure, n (%)	5 (4.31)	6 (4.8)	6 (4.11)	12 (6.9)	2 (1.4)	31 (4.4)	0.221
Peripheral vascular disease, n (%)	0 (0)	1 (0.8)	4 (2.74)	5 (2.87)	6 (4.2)	16 (2.27)	0.151
Cerebrovascular disease, n (%)	2 (1.72)	5 (4)	6 (4.11)	2 (1.15)	6 (4.2)	21 (2.98)	0.346
Dementia, n (%)	0 (0)	0 (0)	0 (0)	0 (0)	0 (0)	0 (0)	NA
Rheumatoid disease, n (%)	1 (0.86)	5 (4)	2 (1.37)	2 (1.15)	3 (2.1)	13 (1.85)	0.340
Peptic ulcer disease, n (%)	1 (0.86)	2 (1.6)	2 (1.37)	1 (0.57)	1 (0.7)	7 (0.99)	0.889
Mild, moderate or severe liver disease, n (%)	5 (4.31)	4 (3.2)	10 (6.85)	26 (14.94)	16 (11.19)	61 (8.66)	0.001
Diabetes and diabetes with complications, n (%)	9 (7.76)	12 (9.6)	13 (8.9)	16 (9.2)	9 (6.29)	59 (8.38)	0.859
Hemiplegia or paraplegia, n (%)	1 (0.86)	0 (0)	0 (0)	2 (1.15)	2 (1.4)	5 (0.71)	0.491
Renal disease, n (%)	4 (3.45)	2 (1.6)	1 (0.68)	4 (2.3)	2 (1.4)	13 (1.85)	0.537
Cancer and metastatic solid tumor, n (%)	3 (2.59)	0 (0)	2 (1.37)	7 (4.02)	2 (1.4)	14 (1.99)	0.137
AIDS/HIV, n (%)	0 (0)	0 (0)	1 (0.68)	0 (0)	0 (0)	1 (0.14)	0.430
Pulmonary hypertension, n (%)	16 (13.79)	23 (18.4)	24 (16.44)	27 (15.52)	28 (19.58)	118 (16.76)	0.737
Hemodialysis, n (%)	5 (4.31)	9 (7.2)	8 (5.48)	10 (5.75)	11 (7.69)	43 (6.11)	0.794
Extracorporeal membrane oxygenation, n (%)	12 (10.34)	12 (9.6)	20 (13.7)	38 (21.84)	33 (23.08)	115 (16.34)	0.002
Tracheostomy, n (%)	22 (18.97)	24 (19.2)	33 (22.6)	33 (18.97)	20 (13.99)	132 (18.75)	0.466

**Table 3:** Complications and pathogen isolations on hospitalized patients with COPD who underwent lung transplantation in Spain from 2016 to 2020.

	2016	2017	2018	2019	2020	TOTAL	*p*-Value Trend
Lung transplant rejection, n (%)	21 (18.1)	18 (14.4)	44 (30.14)	61 (35.06)	26 (18.18)	170 (24.15)	<0.001
Lung transplant failure, n (%)	5 (4.31)	9 (7.2)	17 (11.64)	14 (8.05)	8 (5.59)	53 (7.53)	0.189
Lung transplant infection, n (%)	11 (9.48)	14 (11.2)	24 (16.44)	21 (12.07)	24 (16.78)	94 (13.35)	0.297
Other complications of lung transplant, n (%)	13 (11.21)	9 (7.2)	22 (15.07)	22 (12.64)	14 (9.79)	80 (11.36)	0.311
Unspecified complication of lung transplant, n (%)	6 (5.17)	15 (12)	4 (2.74)	0 (0)	3 (2.1)	28 (3.98)	<0.001
Any complication of lung transplant, n (%)	44 (37.93)	53 (42.4)	83 (56.85)	95 (54.6)	59 (41.26)	334 (47.44)	0.002
Pneumonia, n (%)	5 (4.31)	6 (4.8)	13 (8.9)	13 (7.47)	8 (5.59)	45 (6.39)	0.493
Ventilator associated Pneumonia, n (%)	2 (1.72)	1 (0.8)	2 (1.37)	2 (1.15)	2 (1.4)	9 (1.28)	0.977
*Staphylococcus* bacteremia, n (%)	7 (6.03)	17 (13.6)	19 (13.01)	17 (9.77)	16 (11.19)	76 (10.8)	0.318
*Streptococcus* bacteremia, n (%)	2 (1.72)	2 (1.6)	2 (1.37)	0 (0)	1 (0.7)	7 (0.99)	0.529
Gram-negative bacteremia, n (%)	4 (3.45)	11 (8.8)	12 (8.22)	16 (9.2)	6 (4.2)	49 (6.96)	0.181
Fungemia, n (%)	2 (1.72)	2 (1.6)	3 (2.05)	1 (0.57)	2 (1.4)	10 (1.42)	0.842
*Pseudomonas aeruginosa*, n (%)	9 (7.76)	7 (5.6)	12 (8.22)	5 (2.87)	12 (8.39)	45 (6.39)	0.210
*Aspergillus*, n (%)	6 (5.17)	1 (0.8)	8 (5.48)	12 (6.9)	7 (4.9)	34 (4.83)	0.185
Candidiasis, n (%)	0 (0)	2 (1.6)	1 (0.68)	0 (0)	2 (1.4)	5 (0.71)	0.350
Cytomegalovirus b, n (%)	0 (0)	5 (4)	7 (4.79)	10 (5.75)	8 (5.59)	30 (4.26)	0.144

**Table 4:** In hospital mortality according to study variables among patients with COPD who underwent a lung transplantation in Spain from 2016 to 2020.

		2016	2017	2018	2019	2020	TOTAL	*p*-Value Trend
Type, n (%)	Single	5 (8.47)	2 (3.51)	6 (16.67)	3 (7.89)	8 (24.24)	24 (10.76)	0.028
	Bilateral	6 (10.53)	8 (11.76)	11 (10)	14 (10.29)	7 (6.36)	46 (9.56)	0.303
Sex, n (%)	Men	7 (8.33)	6 (6.32)	10 (10.2)	8 (6.84)	12 (13.19)	43 (8.87)	0.305
	Women	4 (12.5)	4 (13.33)	7 (14.58)	9 (15.79)	3 (5.77)	27 (12.33)	0.426
Age, mean (SD)		58.82 (11.89)	59.2 (2.39)	56.71 (9.38)	56.18 (15.55)	59.53 (4.6)	57.87 (10.2)	0.86
Age groups, n (%)	<40 years	1 (20)	0 (0)	1 (20)	2 (28.57)	0 (0)	4 (19.05)	0.936
	40–49 years	0 (0)	0 (0)	2 (13.33)	1 (7.69)	0 (0)	3 (5.77)	0.964
	50–59 years	1 (1.92)	4 (7.69)	5 (9.26)	5 (8.2)	6 (12.77)	21 (7.89)	0.073
	≥60 years	9 (16.98)	6 (9.23)	9 (12.5)	9 (9.68)	9 (10.98)	42 (11.51)	0.407
CCI, mean (SD)		0.27 (0.47)	0.5 (0.71)	0.82 (0.73)	0.41 (0.62)	0.53 (0.74)	0.53 (0.68)	0.958
CCI, n (%)	0	8 (9.09)	6 (6.52)	6 (5.77)	11 (10.28)	9 (8.91)	40 (8.13)	0.664
	≥1	3 (10.71)	4 (12.12)	11 (26.19)	6 (8.96)	6 (14.29)	30 (14.15)	0.907
Any complication of lung transplant, n (%)	Yes	7 (15.91)	5 (9.43)	11 (13.25)	10 (10.53)	12 (20.34)	45 (13.47)	0.506
	No	4 (5.56)	5 (6.94)	6 (9.52)	7 (8.86)	3 (3.57)	25 (6.76)	0.764

CCI: Charlson comorbidity index.

**Table 5:** Multivariable analysis of the factors associated with in hospital mortality among COPD patients after a lung transplantation in Spain, 2016–2020.

		OR (CI 95%)
Sex	Men	1
	Female	1.59 (0.93–2.72)
Age groups, years	<40	1
	40–49	0.2 (0.04–1.01)
	50–59	0.35 (0.11–1.18)
	≥60	0.56 (0.17–1.8)
CCI index	0	1
	≥1	1.82 (1.08–3.05)
Any complication of lung transplant	No	1
	Yes	2.14 (1.27–3.6)
Year		1.01 (0.83–1.22)

CCI: Charlson comorbidity index; OR: Odds Ratio.

## Data Availability

According to the contract signed with the Spanish Ministry of Health and Social Services, which provided access to the databases from the Spanish National Hospital Database (RAE-CMBD, Registro de Actividad de Atención Especializada. Conjunto Mínimo Básico de Datos, Registry of Specialized Health Care Activities. Minimum Basic Data Set), we cannot share the databases with any other investigator, and we have to destroy the databases once the investigation has concluded. Consequently, we cannot upload the databases to any public repository. However, any investigator can apply for access to the databases by filling out the questionnaire available at https://www.sanidad.gob.es/estadEstudios/estadisticas/estadisticas/estMinisterio/SolicitudCMBD.htm (accessed on 20 October 2022). All other relevant data are included in the paper.
